# Analysis of Fluorescent Carbon Nanodot Formation during Pretzel Production

**DOI:** 10.3390/nano14131142

**Published:** 2024-07-03

**Authors:** Dávid Semsey, Duyen H. H. Nguyen, Gréta Törős, Arjun Muthu, Safa Labidi, Hassan El-Ramady, Áron Béni, Mahendra Rai, Prokisch József

**Affiliations:** 1 Institute of Animal Science, Biotechnology and Nature Conservation, Faculty of Agricultural and Food Sciences and Environmental Management, University of Debrecen, Böszörményi Street 138, 4032 Debrecen, Hungary; semi@gmail.hu (D.S.); toros.greta@agr.unideb.hu (G.T.); labidisafa28@gmail.com (S.L.); hassan.elramady@agr.kfs.edu.eg (H.E.-R.); mahendrarai7@gmail.com (M.R.); jprokisch@agr.unideb.hu (P.J.); 2 Doctoral School of Nutrition and Food Science, University of Debrecen, Böszörményi Street 138, 4032 Debrecen, Hungary; arjun.muthu@agr.unideb.hu; 3 Tay Nguyen Institute for Scientific Research, Vietnam Academy of Science and Technology, 118 Xo Viet Nghe Tinh Street, Da Lat 70072, Vietnam; 4 Doctoral School of Animal Husbandry, University of Debrecen, Böszörményi Street 138, 4032 Debrecen, Hungary; 5 Institute of Agricultural Chemistry and Soil Science, Faculty of Agricultural and Food Sciences and Environmental Management, University of Debrecen, Böszörményi Street 138, 4032 Debrecen, Hungary; beniaron@agr.unideb.hu; 6 Soil and Water Department, Faculty of Agriculture, Kafrelsheikh University, Kafr El-Sheikh 33516, Egypt; 7 Nanobiotechnology Laboratory, Department of Biotechnology, Sant Gadge Baba Amravati University, Amravati 444602, MN, India

**Keywords:** carbon dots, bakery, Maillard reaction, baking, factorial design

## Abstract

Baked pretzels are a popular choice for a quick snack, easily identifiable by their classic twisted shape, glossy exterior, and small salt crystals sprinkled on top, making them a standout snack. However, it is not commonly known that compounds with fluorescent properties can be formed during their production. Carbon nanodots (CNDs) with an average size of 3.5 nm were isolated and identified in bakery products. This study delved into the formation of CNDs in pretzel production using a fractional factorial experimental design. The research revealed that the baking temperature had the most significant impact on the concentration of CNDs, followed by the concentration of NaOH in the immersion solution, and then the baking time. This study highlights the unique role of the NaOH immersion step, which is not typically present in bread-making processes, in facilitating the formation of CNDs. This discovery highlights the strong correlation between the formation of CNDs and the heat treatment process. Monitoring and controlling these factors is crucial for regulating the concentration of CNDs in pretzel production and understanding nanoparticle formation in processed foods for food safety.

## 1. Introduction

Bakery products, such as bread, cake, biscuits, and crackers, are popular worldwide due to their high nutritional value [1,2,3], relatively low cost, and delicious taste. Wheat flour and drinking water are the two main ingredients required to produce bakery products. Pretzels are a famous European bakery product with a knot shape made from wheat flour and water and dipped into NaOH solution [4]. In addition, pretzels contain salt and yeast and incorporate fat, malt extract, starch, and other additives in smaller quantities to achieve the desired product quality [5,6]. Pretzels, characterized by their shiny, dark surface and distinctive flavor, undergo a reaction facilitated by high temperatures and an alkaline environment. This process occurs during pretzel preparation: after shaping, they are submerged in an alkaline solution (typically containing 1–2 m/m% NaOH) and subsequently baked at temperatures around 200 °C [5,6].

The Maillard reactions (MRs) are key processes in forming flavors and colors in bakery products, especially pretzels [7]. The MRs, known as non-enzymatic browning, represent a complex chemical reaction sequence in which amino groups react with carbonyl groups at high temperatures [6,8]. The formation of MRs can be controlled by monitoring baking conditions [9,10,11]. Several by-products of MRs have been reported over the years, including aroma, melanoid compounds, and, recently, carbon nanodots (CNDs)—fluorescent carbon nanoparticles [12,13]. Furthermore, melanoidin, a by-product of CNDs, might be converted into CNDs [14].

Carbon nanodots (CNDs) are carbon nanoparticles that exhibit strong fluorescence, are non-toxic, and are smaller than 10 nanometers in size [15]. These particles have been identified in bakery products [16]. Their fluorescence properties are influenced by their size, surface groups, and defects [17]. CNDs have been detected in various food items, such as caramelized sugar, bread [16], pizza, and beer [18], but their presence in salted pretzels has not yet been investigated. Furthermore, CNDs possess unique fluorescent properties that create opportunities for potential applications such as detecting toxic heavy metals, therapeutic uses, bioimaging, biosensing, and drug delivery [17,18]. Though bread was used as precursor to synthesize CNDs [19], the process of CND formation and the factors influencing it had not been studied previously. Therefore, it is crucial to understand the relationship between CND formation, baking conditions, and their impact on the quality of bakery products.

Therefore, this article examines (1) the fluorescent CNDs formed during pretzel production using fractional factorial experimental design and then (2) seeks to identify correlations between the recipes, manufacturing parameters, and the quantity and quality of fluorescent compounds.

## 2. Materials and Methods

### 2.1. Materials

This study used only food-grade ingredients to prepare the pretzel samples, including BL-55 wheat flour (Hajdú Gabona Zrt., Debrecen, Hungary), tap water, liquid barley malt extract (Ireks Stamag Kft., Komárom, Hungary), native corn starch (m-GEL Hungary Kft., Budapest, Hungary), dried baker’s yeast (König Units Kft., Balmazújváros, Hungary), iodized salt (AGRANA Zucker GmbH, Wien, Austria), granulated sugar (AGRANA Zucker GmbH, Wien, Austria), high oleic sunflower oil (chemiekontor.de GmbH, Mannheim, Germany), sodium metabisulfite (Donauchem Kft., Budapest, Hungary), L-cysteine (Vital-Trend Kft., Budapest, Hungary), sodium hydroxide (AGRANA Zucker GmbH, Wien, Austria), and sugarcane molasses (AGRANA Zucker GmbH, Wien, Austria). All experiments were conducted at Felföldi Confectionery Ltd., Debrecen, Hungary, with 10 repetitions.

### 2.2. Experimental Design

**Figure 1:**
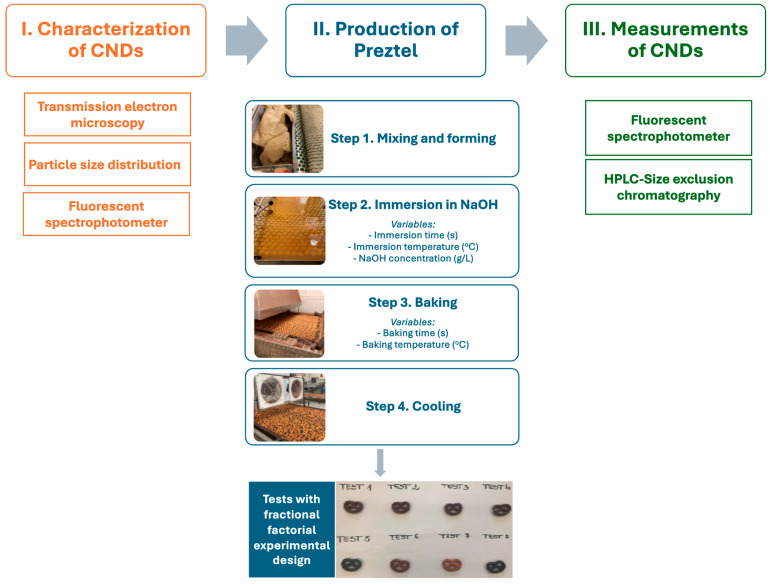
Schematic diagram of the experimental design for studying carbon nanodots (CNDs) formation in pretzel production, including the following: I. Characterization of CNDs in bakery products. II. Pretzel production with fractional factorial experimental design, including four main steps: (1) mixing and forming the dough, (2) immersion of formed pretzel in NaOH solution, (3) baking, and (4) cooling. III. Measurement of CNDs using fluorescent spectrophotometer.

Figure 1 illustrates the experimental design used to study the formation of CNDs in pretzel production. First, CNDs were isolated from bakery products and characterized with TEM and fluorescent spectrophotometer. Then, the fractional factorial experimental design was applied to reduce the number of experiments in pretzel production, which were formulated according to the tests described in Table 1. Five variables were studied in this work: (1) immersion time in NaOH solution (s), (2) temperature of NaOH solution (°C), (3) concentration of NaOH solution (g/L), (4) baking time (min), and (5) baking temperature (°C). Two levels (low and high) were set for each factor based on good pretzel manufacturing practices and experiences, as summarized in Table 2. The eight different settings were recorded to maintain orthogonality during the tests. As part of the experiment, we employed a standard setting (Test 7) commonly utilized in pretzel manufacturing, which could serve as a robust baseline for monitoring changes in fluorescence intensity. All values were given to four significant digits.

CND content in samples was extracted following the published methods with modifications [13]. The pretzel samples were ground into a fine powder and then mixed with ion-exchanged water to prepare a 5 m/m% suspension. To obtain isolated CNDs, the mixture was run through a cellulose column and was filtered through 0.22 µm retention analytical filter paper. Subsequently, the fluorescence of these solutions was measured using a Jasco FP-8500 Spectrofluorometer at the excitation wavelength 370 nm, following the published methods [13]. The isolated CNDs were characterized using a JEM-2000FXII transmission electron microscope (TEM) (JEOL Ltd., Tokyo, Japan).

The water activity of the environment affects the Maillard reaction [7], so we also examined how the water activity of various samples changes depending on different manufacturing parameters and how these are correlated with the fluorescence of the solutions. The water activity of the pretzels was measured using a specialized laboratory-scale water activity meter manufactured by Bionanoferm Ltd., with 5 repetitions.

To determine the size and molar mass of the CNDs, size exclusion chromatography was combined with the fluorescent detector. The following settings were used in the HPLC system (Ecom Spol. Sr.o., Praha, Czech Republic): (1) mobile phase combining between 20% acetonitrile and 80% water; (2) flow rate at 0.7 mL/min; (3) injection volume of 5 μL; (4) Shimadzu RF-20A detector; (5) excitation wavelength at 380 nm and emission wavelength at 460nm. To reduce the hydrophobic interaction inside the column, the above mentioned mobile phase ratio was used [20] and the detector settings were set based on the 3D fluorescent spectra of CNDs.

## 3. Results

### 3.1. Characterization of Carbon Nanodots from Bakery Products

**Figure 2:**
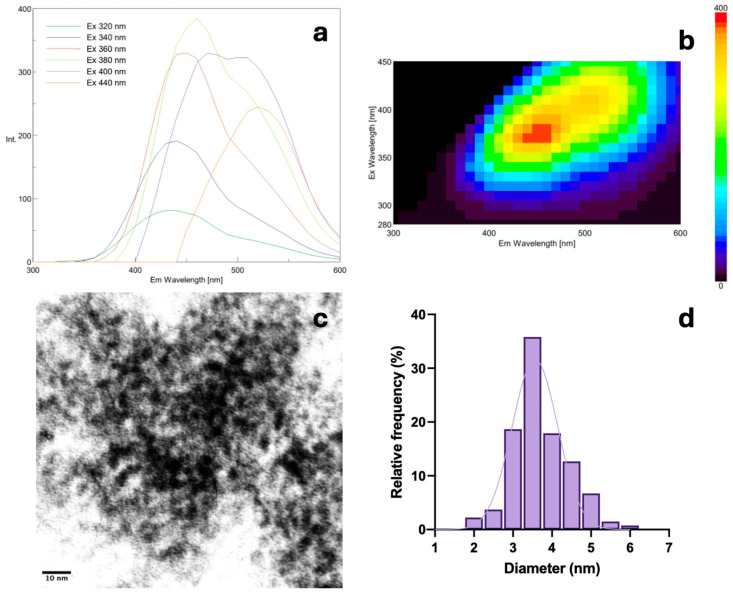
Characterization of carbon nanodots from bakery products (CNDs). (**a**) The emission wavelength 300–600 nm of CNDs with different excitation wavelengths. (**b**) 3D fluorescent spectra of CNDs. (**c**) TEM images of CNDs at the 10 nm scale. (**d**) Size distribution histogram of CNDs with an average size of 3.5 nm.

Carbon nanodots (CNDs) were isolated from pretzels following our published methods [13]. Then, isolated CNDs were characterized with a transmittance electron microscope (TEM) and fluorescent spectrophotometer (Figure 2). Figure 2a,b illustrate CNDs with the optimum excitation wavelength at 380 nm, which slightly changed compared to CNDs obtained from Maillard reactions with the optimum excitation wavelength at 370 nm [13]. CNDs presented with the spere shape in TEM image at 10 nm scale with the recognized average size of 3.5 nm (Figure 2c,d).

### 3.2. Detection of CNDs in Pretzel Production

**Figure 3:**
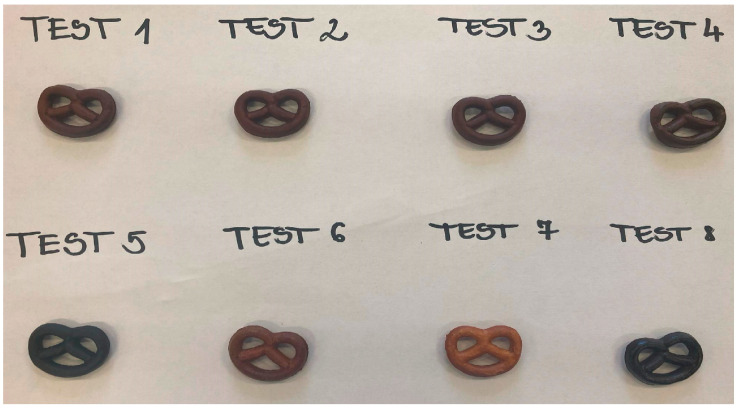
Images of pretzels in different experimental designs after baking.

The diverse effects of various treatments were evident in the external appearances of the specimens, ranging in color from pale brown to deep black, as illustrated in Figure 3. The Test 7 experimental setting, the control setting used for pretzel production, exhibited the faintest hue, consistent with expectations. Remarkable changes could be seen by the naked eye when comparing Test 7 with other samples, which may relate to the burning during baking (Figure 3).

**Figure 4:**
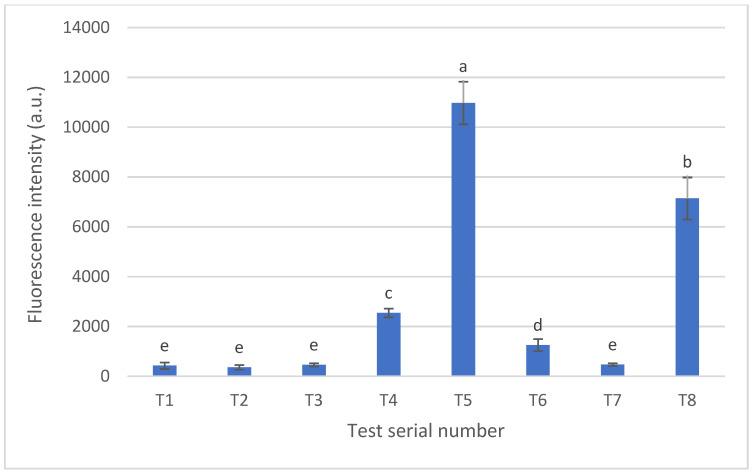
The fluorescent intensity (a.u.) of pretzels at the excitation wavelength of 380 nm. T1–T7 present pretzels at different experimental designs. All samples were measured, with 10 repetitions. Error bars show the standard deviation. Letter a–e indicate the significant difference (*p* < 0.05).

After baking, CNDs were extracted from the pretzel and measured with a fluorescence spectrophotometer at the excitation wavelength of 380 nm, which was observed with CNDs in Figure 2. The fluorescence intensity of baked pretzels is illustrated in Figure 4, whereas sample 5 resulted in the highest intensity with 10,996 ± 854.15 a.u., followed by sample 8 with 7134.7 ± 842.12 a.u. Interestingly, samples T5 and T8 both exhibited a dark color and burnt taste after baking.

**Figure 5:**
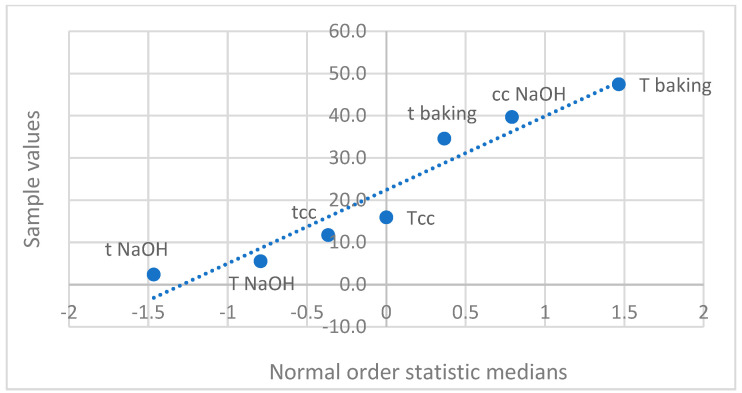
Examination of normal distribution of independent variables. t NaOH: immersion time in NaOH solution (s); T NaOH: immersion temperature in NaOH solution (°C); t baking: baking time (min); T: baking temperature (°C).

**Figure 6:**
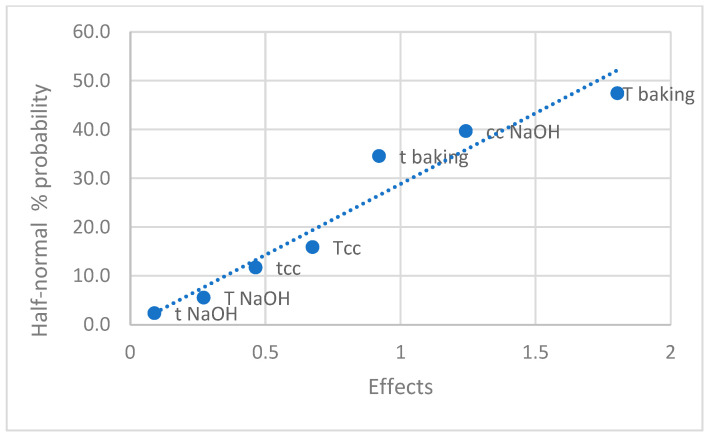
Examination of half-normal distribution of the effects.

The normal distribution of independent variables is illustrated in Figure 5, while the half-normal distribution is presented in Figure 6. If the effects were merely normally distributed random errors, the depicted points would fall onto the line. Figure 5 and Figure 6 show that some effect values do not fall on the line, indicating their significance. The data reveal that the greatest effect on fluorescence intensity, presumably associated with the quantity of CNDs, is exerted by baking temperature. This is followed by the concentration of sodium hydroxide solution and baking time in the order of importance. Compared to the standard sample T7, sample T5 exhibited a fluorescence intensity 23.6 times greater, experiment 8 showed a 15.36-fold increase, and experiment 4 displayed a 5.46-fold increase. Notably, unlike experiment 7, where the baking temperature was set to 200 °C, these experiments also underscore the essential role of baking temperature in forming CNDs.

### 3.3. Carbon Nanodot Concentration (mg/kg) in Pretzel Production

**Figure 7:**
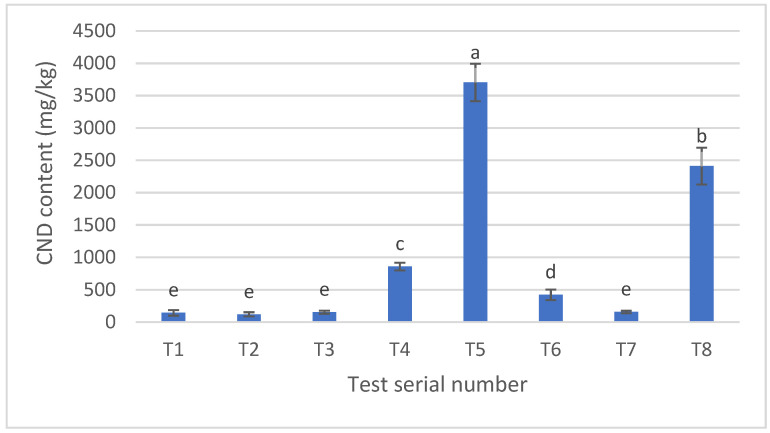
Carbon nanodot concentrations (mg/kg) of the pretzel samples. T1–T7 present pretzels at different experimental designs. All samples were measured, with 10 repetitions. Error bars show the standard deviation. Letters a–e indicate the significant difference (*p* < 0.05).

Following a previous study, we further investigated the possibility of correlating the measured fluorescence intensity to the concentration of CNDs [13]. To achieve this, a standard containing CNDs was created, and its fluorescence, carbon, and nitrogen content were analyzed. These measurements were then used to establish a calibration curve. As a result, the proportionality factor between fluorescence intensity and CND concentration was determined to be 0.3377. The data obtained from these measurements is depicted in Figure 7. Notably, samples T5 and T8, which exhibited a dark color during baking (as shown in Figure 3), displayed the highest CND content (mg/kg). This finding strongly suggests a relationship between the formation of CNDs and the heat treatment process, similar to the previous studies [13,16,18]. Conversely, sample T7, prepared using a recipe commonly used for commercialized products, demonstrated a low concentration of CNDs.

**Figure 8:**
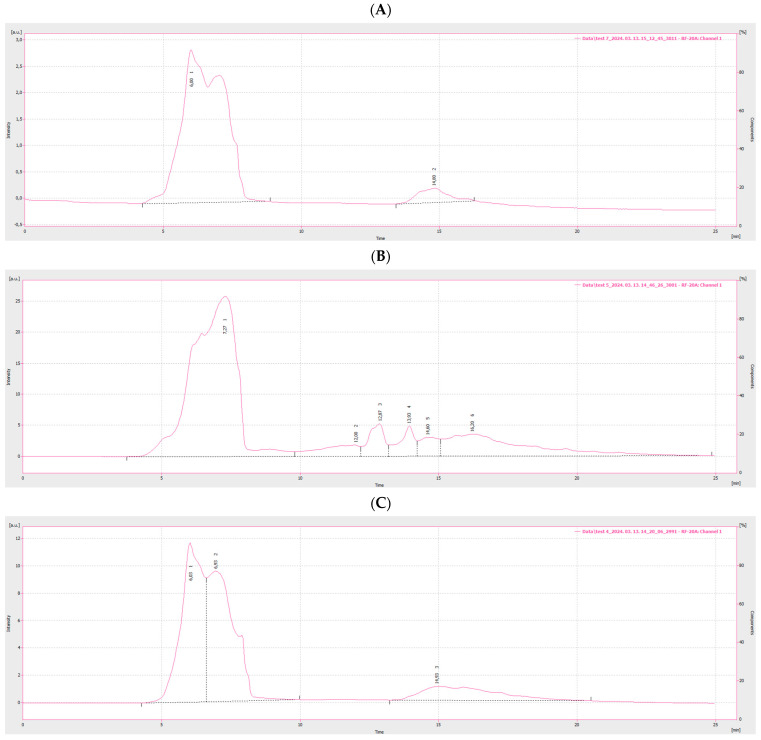
HPLC- size exclusion chromatogram of pretzel samples. (**A**) Sample T7. (**B**) Sample T5. (**C**) Sample T4.

To differentiate between the three samples T7, T4, and T5, an analysis was conducted using size exclusion chromatography (SEC) with a fluorescent detector connected to the HPLC system. The resulting chromatograms can be seen in Figure 8. CNDs were observed at the first peak with the highest fluorescent intensity, consistent with the results shown in Figure 7. Sample T5 exhibited the highest intensity, followed by sample T4, and the lowest intensity was observed in sample T7. Based on the molecular mass of the first peak, the diameter of CNDs was calculated in three samples including approximately 3 nm for sample T7, followed by 2 nm for sample T5 and 3 nm for sample T4.

### 3.4. Water Activity in Baked Pretzels

**Figure 9:**
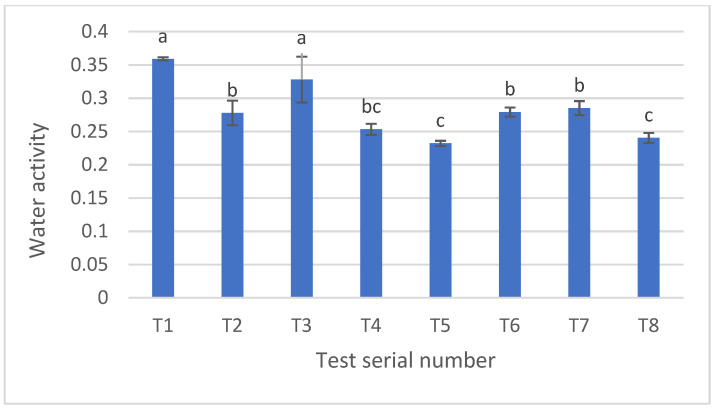
Water activities of the baked pretzels. T1–T7 presents pretzels at different experimental designs. All samples were measured, with 10 repetitions. Error bars show the standard deviation. Letter a–c indicate the significant difference (*p* < 0.05).

The average water activities of the baked pretzels ranged between 0.23 and 0.36, with the three lowest values associated with the three samples showing the highest fluorescence intensity. While a direct proportionality cannot be conclusively inferred, it appears that pretzels roasted at higher temperatures for longer durations exhibited lower water activity values. The means and deviations of water activities are depicted in Figure 9.

## 4. Discussion

Although carbon nanodots (CNDs) were reportedly present in bread [13,16], there is no report on the presence of these nanoparticles in pretzel production. This study aimed to understand the formation of CNDs in the production of pretzels through factorial experimental design. Initially, the isolation and characterization of carbon nanodots (CNDs) in bakery products (TEM and fluorescent spectrophotometer) have proven the presence of CNDs in pretzels. CNDs show the highest fluorescent intensity at the excitation wavelength of 380 nm and an average size of 3.5 nm (Figure 2). Therefore, 380 nm is chosen as the excitation wavelength for all measurements.

This work studied five independent variables with a factorial experimental design, including (1) immersion time in NaOH solution (s), (2) temperature of NaOH solution (⁰C), (3) concentration of NaOH solution (g/L), (4) baking time (min), and (5) baking temperature (⁰C) with two adjusted levels (Table 2). The fluorescent intensity of CNDs in eight samples was isolated and measured. Notably, the highest level of CNDs was found in sample T5 and sample T8 (Figure 4), which also exhibited a black color and burnt taste after baking (Figure 3). This result was similar to the previous studies that reported the close relation between the formation of CNDs and heat treatment products [16,18]. Furthermore, after analyzing the results, the CND content of the examined baked pretzel samples was influenced by three primary factors: baking temperature, the concentration of the dipping alkaline solution, and baking time. Elevating these factors to high levels resulted in an increase in fluorescence, suggesting a corresponding increase in the quantity of CNDs. It could be noted that monitoring these factors could control the formation of CNDs in production. Furthermore, the rapid detection of over-baking pretzels could be monitored by measuring the CND content in the samples.

CNDs have never been investigated in baked pretzels, and the fluorescence of solutions made from them has not been measured either. Thus, exploring this avenue may yield many novel findings. The next step is to study whether there is indeed a presence of CNDs in foods and, if so, what their significance may be. If their beneficial effects are confirmed, we need to understand how to achieve high concentrations of CNDs without adversely affecting the sensory properties of the product. Human clinical trials are already underway regarding the beneficial effects of CNDs on the human body; once these trials yield certainty, this research will take on new significance, potentially opening a new chapter in the healthy snacking category. These trials are pivotal in establishing the efficacy and safety profile of CNDs for human consumption. The outcomes of these trials will not only validate the potential health benefits but also enhance the credibility and applicability of our findings in real-world settings.

## 5. Conclusions

In summary, pretzels were subjected to various controlled treatments, and then the extent to which these interventions affected the fluorescence of the samples was examined. Following an HPLC analysis, it was found that there was a direct correlation with CND concentrations. The baking temperature had the greatest impact, with the highest CND concentration values associated with samples treated at higher temperatures. In addition to baking temperature, the concentration of NaOH in the immersion solution and the baking time also had significant effects. From the water activity tests, it was revealed that samples exhibiting higher fluorescence values corresponded to the lowest water activity values.

## Figures and Tables

**Table 1:** Different recipes of pretzel production with fractional factorial experimental design including 8 controlled variables and 5 independent variables.

Ingredients and Factors	Test 1	Test 2	Test 3	Test 4	Test 5	Test 6	Test 7	Test 8
**Controlled variables**								
BL-55 wheat flour quantity (%) (*w*/*w*)	67.92							
Liquid barley malt extract quantity (%) (*w*/*w*)	1.358							
Native corn starch quantity (%) (*w*/*w*)	2.037							
Dried baker’s yeast quantity (%) (*w*/*w*)	0.6792							
Granulated sugar quantity (%) (*w*/*w*)	0.6792							
High oleic sunflower oil quantity (%) (*w*/*w*)	6.112							
Sodium metabisulfite quantity (%) (*w*/*w*)	0.04292							
L-cysteine quantity (%) (*w*/*w*)	0.008557							
**Independent variables**								
Immersion time in NaOH solution (s)	5	20	5	20	5	20	5	20
Temperature of NaOH solution (°C)	40	40	80	80	40	40	80	80
Concentration of NaOH solution (g/L)	10	10	10	10	30	30	30	30
Baking time (min)	10	20	20	10	20	10	10	20
Baking temperature (°C)	240	200	200	240	240	200	200	240

**Table 2:** Variables and levels used in fractional factorial experimental to study the formation of carbon nanodots (CNDs) in pretzel production.

Variable	Level	
	Low (−1)	High (+1)
Immersion time in NaOH solution (s)	5	20
Temperature of NaOH solution (°C)	40	80
Concentration of NaOH solution (g/L)	10	30
Baking time (min)	10	20
Baking temperature (°C)	200	240

## Data Availability

The raw data supporting the conclusions of this article will be made available by the authors on request.
